# Prone Position and Cardiopulmonary Resuscitation in the Operating Room: A Scoping Review

**DOI:** 10.3390/jcm14062044

**Published:** 2025-03-17

**Authors:** Eleonora Case, Corina Elena Luca, Paolo Maino, Claudio Speroni, Giovanna Pezzoli, Matteo Gianinazzi, Loris Bonetti

**Affiliations:** 1 Department of Nursing, Regional Hospital of Lugano, Ente Ospedaliero Cantonale (EOC), 6900 Lugano, Switzerland; eleonora.case@eoc.ch (E.C.); claudio.speroni@eoc.ch (C.S.); giovanna.pezzoli@eoc.ch (G.P.); 2 Department of Anaesthesiology, Intensive Care and Emergency Medicine, Regional Hospital of Lugano, Ente Ospedaliero Cantonale (EOC), 6900 Lugano, Switzerland; paolo.maino@eoc.ch (P.M.); matteo.gianinazzi@edu.ti.ch (M.G.); 3 Faculty of Biomedical Sciences, Università della Svizzera Italiana, 6900 Lugano, Switzerland; 4 Scuola Specializzata Superiore in Cure Infermieristiche, 6928 Manno, Switzerland; 5 Nursing Research Competence Centre, Department of Nursing, Ente Ospedaliero Cantonale (EOC), 6500 Bellinzona, Switzerland; loris.bonetti@eoc.ch

**Keywords:** cardiopulmonary resuscitation, prone positioning, operating room, defibrillation

## Abstract

**Background/Objectives:** Cardiopulmonary resuscitation (CPR) in the prone position (P-CPR) is described in international guidelines for specific contexts but is not commonly included in operating room algorithms. This review aims to map P-CPR interventions in adult and pediatric patients experiencing cardiac arrest in the operating room while in the prone position. **Methods:** A scoping review was conducted following the “PRISMA Extension for Scoping Reviews” protocol. The databases searched included PubMed, CINAHL, ScienceDirect/Elsevier, Scopus, Web of Science, and Cochrane. Eligibility criteria included studies involving adult and pediatric populations, documented cardiac arrest (with presenting rhythm and cause), P-CPR interventions, and short-term outcomes (return of spontaneous circulation) as well as long-term outcomes when available. **Results:** Twenty international case reports were analyzed, indicating that P-CPR is effective in the operating room setting and has a positive impact on both short-term and long-term outcomes. **Conclusions:** This scoping review suggests that P-CPR yields comparable outcomes to supine CPR while saving time by eliminating the need for patient repositioning. However, due to limited evidence, further research is needed. Additionally, logistical, organizational, and educational considerations must be addressed before adopting P-CPR as routine practice.

## 1. Introduction

Cardiac arrest in the operating theater is a rare but potentially catastrophic event, with mortality rates exceeding 50% [1]. The causes of intraoperative cardiac arrest are usually known, and because patients are continuously monitored, these events are typically recognized early.

Unlike other clinical settings, intraoperative cardiac arrest is influenced by preoperative patient conditions as well as surgical and anesthetic factors, including hypoxia, hemorrhagic shock, pulmonary embolism, myocardial infarction, arrhythmias, and electrolyte disturbances [1,2,3]. According to the American Society of Anesthesiologists (ASA), predictive factors for intraoperative cardiac arrest include sepsis, the urgency of the procedure, the type of surgery, the anesthetic technique used, and the patient’s age [1,4]. The 2021 European Resuscitation Council (ERC) Guidelines recommend a tailored approach to cardiac arrest in the operating room, leveraging continuous patient monitoring and a multidisciplinary team trained to manage perioperative emergencies [5]. The incidence of perioperative cardiac arrest is highest in children, particularly infants, and older patients [6].

Intraoperative cardiac arrest is a serious complication of non-cardiac surgery, with an immediate survival rate of 50%. Managing such events is particularly challenging for surgeons and anesthetists, especially during elective procedures [1]. These events are almost always witnessed, requiring anesthesiologists to simultaneously diagnose and treat the underlying causes while leading a multidisciplinary resuscitation effort [7].

Cardiac arrest can also occur in patients in the prone position, particularly in interventional radiology and surgical procedures. This positioning can contribute to cardiovascular instability due to hypovolemia, reduced preload, and possible right and left ventricle dysfunction, increasing the risk of cardiogenic shock [8]. Performing cardiopulmonary resuscitation (CPR) on a prone patient presents additional challenges, as it is not a widely known or routinely practiced technique [9].

Prone cardiopulmonary resuscitation (P-CPR) is an alternative resuscitation method applied in cases of cardiac arrest occurring while the patient is in the prone position [10]. While conventional CPR is performed in the supine position, certain clinical scenarios—such as neurosurgical and spinal procedures—may necessitate resuscitation without repositioning the patient [10]. Repositioning from prone to supine delays the initiation of CPR, with studies indicating that the supination maneuver can take at least five minutes and requires three to five operators to execute safely [11].

Additionally, performing CPR in the prone patient is complicated by the surgical environment, which may involve open wounds exposing critical anatomical structures such as the brain or spine, protruding metal instruments (e.g., in endoscopic or robotic neurosurgery), and specialized skull fixation systems. Although international guidelines recommend P-CPR, timely intervention remains a critical factor in its effectiveness [12]. However, no standardized algorithm currently exists for P-CPR in the operating room setting.

## 2. Methods

### 2.1. Design

This scoping review was conducted following the “PRISMA Extension for Scoping Reviews (PRISMA-ScR): Checklist and Explanation” [13].

### 2.2. Aim

The aim of this study is to map P-CPR interventions in adult and pediatric patients who experience cardiac arrest in the operating room while in a prone position.

### 2.3. Inclusion and Exclusion Criteria

Studies were included if they were published in English, German, French or Italian, were conducted in the operating room setting, and were available in full-text format. Eligibility criteria were structured according to the patient–intervention–comparison–outcome (PICO) framework:

○ Population: adult or pediatric patients experiencing cardiac arrest onset in the operating room;
○ Intervention: P-CPR;
○ Comparison: standard care (supine position); and
○ Outcome: return of spontaneous circulation (ROSC) or death;

Studies focusing on intensive care or pre-hospital settings were excluded. Other exclusion criteria were abstracts of articles not fully searchable and conference proceedings. No restrictions were placed on study quality or design, following the “pyramid of evidence” framework [14].

### 2.4. Databases Used and Search Period

The following databases were searched: PubMed, CINAHL, Cochrane, Scopus, Web of Science, and ScienceDirect/Elsevier (Supplementary File S1). Additionally, gray literature was explored using Google to identify the latest American Heart Association (AHA) guidelines on CPR in specific conditions [10]. The initial search was completed in January 2023, with an update in July 2024 that yielded no additional records.

### 2.5. Source Selection Process

The PRISMA-ScR protocol flowchart was used to document the source selection process [13]. Articles identified through database searches were uploaded to Rayyan (http://rayyan.qcri.org, last accessed 31 July 2024), a free web-based tool and mobile app used for streamlining article screening. Rayyan was used throughout all screening and selection stages.

Two authors (EC and ECL) independently performed data selection, with discrepancies resolved through consultation with a third author (PM). Data extraction was also conducted independently by EC and ECL, with consensus reached through discussion when necessary.

### 2.6. Data Extraction and Synthesis

Qualitative and quantitative data were extracted from selected studies, focusing on key study characteristics and statistical variables. The results were summarized in a table consisting of seven items: (1) author, year, and country of publication; (2) study design and title; (3) context (type of surgery and setting); (4) patient characteristics, including age and sex; (5) event and cause, including confirmed cardiocirculatory arrest (CA) and invasive ventilation. This included defibrillable rhythms (i.e., pulseless ventricular tachycardia [VT], ventricular fibrillation [VF]) and non-defibrillable rhythms for which there is no indication for the use of a defibrillator non-defibrillable rhythms (i.e., pulseless electrical activity [PEA], asystole); (6) intervention, including CPR and defibrillation (if indicated), in the prone position; and (7) outcome (ROSC or death, intensive care unit [ICU] discharge, follow-up, and neurological status when available).

The content analysis results were first summarized by describing the study designs, primary interventions, and the geographic distribution of cases. The effectiveness of P-CPR and its role in different types of cardiac arrest were then described.

### 2.7. Critical Appraisal of Sources

As this was a scoping review, a formal systematic review with critical appraisal was not conducted. However, a detailed analysis of the available data was performed to address the study objective.

## 3. Results

### 3.1. Selection of Studies

**Figure 1:**
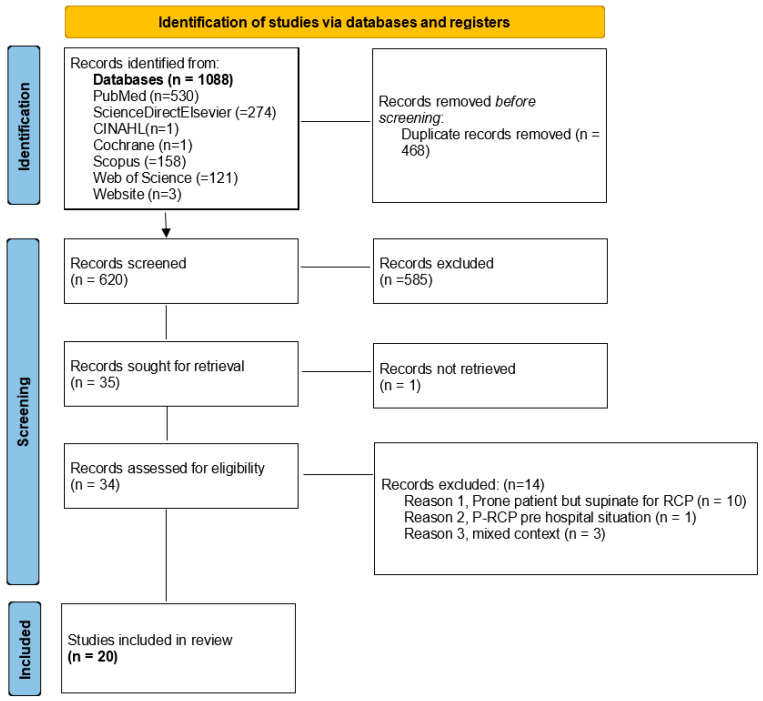
PRISMA-ScR flowchart.

The initial search of electronic databases yielded 1088 articles, 468 of which were eliminated as duplicates. The remaining 620 articles were screened by title and abstract, resulting in 34 records selected for eligibility. Of these, 14 articles were excluded for the following reasons: supination of the patient during CPR, a mixed operating room (OR) and ICU context, pre-hospital settings without advanced airway management, or inclusion of the lateral position. This resulted in a total of 20 eligible articles (Figure 1).

### 3.2. Description of Included Studies

The characteristics of the included studies are summarized in Table 1. The dataset comprises 20 case reports involving 22 patients and a total of 25 cardiac arrest episodes. Each case report included a single patient [9,10,15,16,17,18,19,20,21,22,23,24,25,26,27,28,29,30,31], except for one case, which described two patients [32]. All cases met the predefined selection criteria, took place exclusively in the OR, and involved patients who had previously achieved advanced airway management. The studies span from 1982 to 2020.

Nine cranial surgeries [16,17,19,20,22,28], 12 spinal surgeries [9,15,18,21,24,25,26,27,32], and one orthopedic/traumatologic surgery [23] were considered. The patient cohort consisted of 11 adults (six men and five women) aged 28 to 81 years and 11 children ranging in age from six months to 15 years.

### 3.3. Summary of Results—Outcomes of Prone Resuscitation in the Included Case Reports

This section reports the conditions that led to the need for CPR and the recorded outcomes in the studies on P-CPR.

The rhythm associated with CA varied across studies. One case involved an unspecified CA [31]. Regarding non-defibrillable rhythms, there were eight episodes of PEA [9,15,17,18,20,23,30,32], one of which progressed to asystole [27], and nine cases of asystole [10,21,22,27,28,29,32]. Among defibrillating rhythms, there were five cases of VF [16,19,24,26,30] and three cases of pulseless VT [16,19,25].

The primary causes of CA included gas embolism (seven cases) [9,18,23,25,28,32], massive hemorrhage (five cases) [10,17,20,28,29], hypoxia secondary to endotracheal tube obstruction (one case) [30], myocardial ischemia (one case) [27], cardiac tamponade (one case) [15], myocardial insufficiency (one case) [22], and electrolyte imbalance with hyperkalemia (one case) [24].

CPR was performed exclusively in the prone position, achieving ROSC in all cases. In four cases, supination was used to continue the resuscitative maneuvers after initial P-CPR [9,15,27,32]. Defibrillation was performed in six of the cases requiring it [16,19,24,25,26,30]. In one case, defibrillation was not performed despite a defibrillable rhythm, as the primary cause of CA (hypoxia due to endotracheal tube obstruction) was resolved [30]. Defibrillator pads were placed on the lower back along the mid-axillary line and just below the right scapula [24]. In one case, direct defibrillation and internal cardiac massage were performed via posterior thoracotomy due to spinal instability and the presence of metallic implants preventing movement [26].

The outcome included two intraoperative deaths in pediatric patients due to massive embolism, with prior histories of cerebral palsy and muscular dystrophy [32]. P-CPR was initiated but subsequently continued in the supine position in both cases. Two additional cases involved intraoperative ROSC followed by death in the ICU at five and 28 days: one following massive hemorrhage in a six-year-old girl and the other after emergency craniotomy for cerebral hemorrhage with asystole due to probable brainstem compression [17,22].

In the remaining 18 cases, intraoperative ROSC was achieved, followed by ICU admission and eventual discharge with favorable neurological outcomes [9,10,15,16,18,19,20,21,23,24,25,26,27,29,30,31]. The surgical intervention was completed in most cases [9,15,16,18,19,22,24,25,26,29,30,31], while in a few instances, the procedure was postponed [23,27,30].

Short-term and long-term outcomes were analyzed to evaluate the effectiveness of P-CPR in patients experiencing cardiac arrest in the prone position [33]. Short-term outcomes included ROSC and survival until hospital admission. ROSC indicates the immediate success of resuscitation efforts, reflecting restored spontaneous cardiac activity [33]. ICU admission was also considered a relevant short-term outcome, reflecting post-resuscitation stabilization. These criteria align with standard definitions in resuscitation research, which focus on the immediate physiological response to CPR [9,10,15,16,17,18,19,20,21,22,23,24,25,26,27,28,29,30,31,32].

Long-term outcomes were assessed based on survival to hospital discharge, neurological recovery using the Cerebral Performance Categories (CPC) scale, and functional independence at follow-up [33]. Survivors exhibited varying degrees of neurological recovery, highlighting the importance of post-resuscitation care and rehabilitation. Some reports included follow-up data assessing functional recovery beyond hospitalization [15,16,17,18,19,20,23,24,25,26,27,28].

Additionally, at least one case included a follow-up period extending to 24 months, providing valuable insights into sustained neurological and functional outcomes over time. This information is crucial for understanding the long-term impact of P-CPR on patient prognosis [16].

## 4. Discussion

The aim of this scoping review was to map P-CPR interventions in adult and pediatric patients experiencing cardiac arrest in the OR in the prone position.

### 4.1. Effectiveness of P-CPR

Based on the included studies, P-CPR appears to be effective in OR settings, particularly when performed by trained healthcare staff [32]. Selected case reports detailed trends and continuous monitoring of end-expiratory carbon dioxide (ETCO2) and diastolic blood pressure (DBP), key indicators of CPR quality. Among the examined records, diastolic blood pressure was measured invasively in 13 cases [9,15,16,17,20,22,23,24,25,28,29,30,31], non-invasively in 4 cases [18,21,26,27], and was not explicitly specified in 3 cases [10,19,32].

ETCO2 values primarily reflect pulmonary blood flow and cardiac output. Capnometry and waveform capnography analysis can aid in assessing patient status during CPR [34,35]. The 2018 AHA Consensus Statement, endorsed by the American College of Emergency Physicians and the Society of Critical Care Medicine in 2018, provides reference values for ETCO2 and DBP as indicators of resuscitation effectiveness [36]. ETCO2 > 10 mmHg (1.33 kPa) is suggestive of poor cardiac output and strongly predicts resuscitation failure. The optimal ETCO2 value is >20 mmHg (2.66 kPa) with a ventilation rate of 10 breaths per minute. A rapid increase to 35–45 mmHg (4.66–5.99 kPa) signals ROSC.

The European Resuscitation Council (ERC) 2021 and the American Heart Association (AHA) 2023 guidelines, which specify maintaining invasive diastolic arterial pressure (DAP) > 25 mmHg in adults and infants or ≥30 mmHg in older children is associated with higher survival rates [5,37].

### 4.2. Comparison with the Supine Position

The case reports included in this review suggest that P-CPR offers potential advantages, particularly in the timely initiation of resuscitative maneuvers. This is especially relevant in operating theater settings, where patients are under continuous monitoring and their airways are already secured. Furthermore, if chest compressions are effective, interruptions required for supination can be minimized. The importance of optimizing the compression fraction (CCF) by reducing both the number and duration of interruptions between compressions is emphasized in the AHA guidelines [36,37]. Maintaining a CCF value > 80% is considered optimal for ensuring adequate tissue perfusion (CCF = % of time chest compressions are performed that generate blood flow) [36].

Initiating CPR immediately after a loss of cardiac output in a prone patient—rather than waiting for sufficient personnel to safely position them supine—can save time and improve outcomes, as early CPR is associated with better survival rates following cardiac arrest [38].

The rapid initiation of resuscitative measures in the case reports analyzed contributed to the positive outcomes observed in the majority of cases. The absence of delays related to organizing and executing supination maneuvers reduced the time between CA recognition and CPR initiation, allowing for uninterrupted intervention.

Existing theories on CPR mechanics suggest that P-CPR is feasible, as chest compression in both the supine and prone positions generates sufficient force to produce a detectable pulse. Effective compressions should reach a depth of 5–6 cm in adults or one-third of the anterior–posterior chest diameter in pediatric patients. Additionally, current recommendations suggest a compression rate of 100–120 per minute, ensuring adequate chest recoil between compressions [39].

### 4.3. Issues of Clinical Viability

While P-CPR has demonstrated feasibility in case reports, several challenges must be addressed before it can be integrated into standard resuscitation protocols. Various technical adaptations and device modifications have been explored to enhance its effectiveness and safety.

First, unlike conventional CPR, P-CPR requires specific knowledge of compression landmarks, patient positioning, and airway management adaptations. Studies utilizing computerized tomography imaging have identified the optimal compression point at the T8–T9 vertebral level, just below the scapula, which differs from the traditional sternal compression site used in supine CPR [40]. Additionally, alternative compression techniques, such as bilateral paravertebral compressions, have been suggested when direct sternal compression is not feasible due to the surgical field [40]. The absence of standardized training programs may lead to variability in technique and inconsistent effectiveness, highlighting the need for structured educational initiatives.

Furthermore, implementing CPR in real-world clinical settings presents logistical barriers, as exemplified by the case of Miranda and Newton [24]: “*In the case described, the patient was prone with pins screwed into the skull, covered with surgical drapes, and had an open surgical wound on the back of the chest. In addition, numerous intravascular lines and breathing circuitry connected her to the anaesthetic machine and monitors. Turning her into the supine position to carry out defibrillation and cardiopulmonary resuscitation would have taken several minutes. Movement of the now unstable column during this manoeuvre would almost certainly have resulted in spinal cord damage.*”. The presence of surgical drapes, positioning aids, and limited access to the anterior chest wall can complicate compression mechanics and airway management, challenges underscored also by Brown et al. [25] Additionally, in critical care units and ORs, repositioning a patient from prone to supine may introduce delays, raising concerns about “no-flow” time and the risk of secondary injuries. Studies have proposed alternative defibrillation pad placements—such as bilateral mid-axillary positions or a combination of scapular and mid-axillary placement—to optimize electrical current flow and improve shock efficacy [24,25,41].

Currently, no specialized devices have been developed specifically for prone resuscitation, necessitating modifications to existing CPR equipment. Studies have explored the use of mechanical compression devices, such as the AutoPulse™, in prone patients, with some evidence suggesting that compression depth remains within acceptable limits when properly positioned [42]. Additionally, experimental models have demonstrated that placing a sandbag or a firm cushion beneath the sternum may enhance compression depth and improve hemodynamic effectiveness [43]. These challenges highlight the need for further investigation into the clinical viability of P-CPR. Future research should focus on training standardization, protocol optimization, and technological advancements to enhance the safety and effectiveness of this technique. Based on the case reports analyzed, P-CPR appears to be a safe technique that enables timing intervention by medical staff. Early chest compressions are known to significantly increase survival rates [44], supporting the recommendation of this procedure. However, given the absence of higher-quality studies, such as cohort or randomized controlled trials, further research is needed to confirm the effectiveness of P-CPR.

### 4.4. Limitations and Strengths

One limitation of this review is that the current literature consists primarily of case reports, which provide only level 5 evidence. More robust studies, such as prognostic cohort studies, are needed to confirm at least the non-inferiority of prone resuscitation for patients in this position due to specific interventions.

Compared to standard CPR, P-CPR has been reported in far fewer cases. Most existing systematic reviews include patients from various settings, particularly ICU patients treated during the COVID-19 pandemic, which brought attention to this issue worldwide.

The case reports reviewed do not specify whether the personnel performing cardiac massage in the prone position had prior training. Establishing training programs for P-CPR is crucial to prevent delays in initiating CPR due to uncertainty in technique.

It is recommended that cases of prone cardiac arrest in the OR be thoroughly documented, considering both short-term and long-term outcomes. Additionally, the creation of a multicenter registry could facilitate a more in-depth investigation of this topic.

A strength of this review is that, to the best of our knowledge, it is the first to summarize the existing literature on this issue. This may serve as a foundation for further research.

## 5. Conclusions

This scoping review synthesizes the existing literature on the performance of P-CPR in the operating theater. The findings suggest that P-CPR yields outcomes comparable to those of supine CPR while reducing the time required to reposition the patient. These results indicate that the procedure could be integrated into clinical practice in operating theaters and other settings where prone interventional procedures are common.

However, due to the limited quality of the available evidence, further studies are necessary to validate the conclusions drawn in this scoping review. Additionally, logistical, organizational, and educational challenges must be addressed before P-CPR can be implemented as a standard procedure. Once more robust evidence becomes available, developing a standardized algorithm for P-CPR in the prone position would be essential for its integration into clinical practice.

## Figures and Tables

**Table 1:** Characteristics of the included studies.

Author (Year) Country	Study Design & “Title”	Context	Patient	Event and Cause	Intervention	Outcome
Al Harbi et al. (2020) Saudi Arabia [9]	Case report “*Prone cardiopulmonary resuscitation in elderly undergoing posterior spinal fusion with laminectomy*”	Spinal surgery: Posterior spinal fusion (PLIF) with laminectomy	Man 80 Y	Two episodes of PEA at 6 h after induction. Cause: massive pulmonary embolism.	First episode: CPR in the prone position Second episode: CPR in the supine position	First episode: ROSC Second episode: ROSC and admission to ICU.
Mishra et al. (2019) India [15]	Case report “*Cardiac arrest in the prone position caused by central venous cannulation-induced cardiac tamponade*”	Spinal surgery: Laminectomy and excision of an intradural and extramedullary meningioma of C2–C3	Woman 35 Y	PEA caused by cardiac tamponade	One min of CPR in the prone position and then in the supine position	ROSC and admission to ICU with pericardial suction drainage. Discharge after 3 days.
Mayorga-Buiza et al. (2018) Spain [16]	Case report “*Cardiac pulmonary resuscitation in prone position. The best option for posterior fossa neurosurgical patients*”	Cranial surgery: Excision of a large tumor in the posterior cranial fossa	Boy 10 Y	Pulseless VT and subsequent VF during tumor removal	CPR in the prone position and defibrillation.	ROSC followed by surgically drained spinal hematoma. ICU admission and discharge after 5 days, positive follow-up at 24 months.
Burki et al. (2017) Pakistan [17]	Case report “*CPR in prone position during neurosurgery*”	Cranial surgery: Fourth ventricle tumor excision	Girl 6 Y	PEA due to massive hemorrhage (estimated loss 2 L)	CPR in the prone position	ROSC and admission to ICU. Death after 5 days.
Kaloria et al. (2017) India [18]	Case report “*Venous air embolism during removal of bony spur in a child of split cord malformation*”	Cranial surgery: Bone spur removal for “Split spinal cord malformation” (SSCM)	Girl 1 Y	PEA from probable venous gas embolism	CPR in the prone position	ROSC, completion of surgery and admission to ICU. Discharge after 6 days.
Taylor et al. (2013) New Zealand [19]	Case report “*Cardiac arrest during craniotomy in prone position*”	Cranial surgery: Craniotomy for metastatic melanoma	Man 69 Y	Pulseless VT and subsequent VF	Defibrillation and CPR in the prone position	ROSC and admission to ICU. Discharge to home in stable neurological condition.
Gomes et al. (2012) Brazil [20]	Case report “*Cardiopulmonary resuscitation in the prone position*”	Cranial surgery: Excision of very vascularized, parietal-right occipital meningioma.	Woman 77 Y	PEA from sagittal sinus rupture and subsequent hemorrhagic shock	CPR in the prone position	ROSC and admission to ICU. Discharge after 3 days.
Dooney (2010) Australia [21]	Case report “*Prone CPR for transient asystole during lumbosacral spinal surgery*”	Spinal surgery: L4–L5 micro discectomy	Man 43 Y	Asystole	CPR in the prone position	ROSC, ICU admission.
Haffner et al. (2010) Germany [22]	Case report “*Erfolgreiche kardiopulmonale Reanimation in Bauchlage*”	Cranial surgery: Emergency craniotomy for cerebral hemorrhage	Man 81 Y	Asystole due to probable brainstem compression	CPR in the prone position	ROSC, ICU admission, and death at 28 days.
Almazan and Brock-Utne (2009) USA [23]	Case report “*Case Studies of Near Misses in Clinical Anesthesia*”	Orthopedics/traumatology: Internal fixation and reduction in multiple pelvic fractures	Man 28 Y	PEA caused by pulmonary embolism	CPR in the prone position	ROSC, ICU admission. Completion of surgery 3 days later and discharged from hospital without neurological deficit.
Miranda and Newton (2001) UK [24]	Case report “*Successful defibrillation in the prone position*”	Spinal surgery: Palliative debulking of metastatic tumor at T3 level and internal stabilization	Woman 39 Y	VF from electrolyte imbalance. (hyperkalemia)	Precordial thump and CPR in prone position and defibrillation with a single shock at 200 J	ROSC, completion of surgery, and discharge home one week after the event.
Brown et al. (2001) UK [25]	Case report “*Cardiac arrest during surgery and ventilation in the prone position: a case report and systematic review*”	Spinal surgery: Thoracic decompression of invasive tumor from T11 to L1	Woman 60 Y	Wide complex tachycardia followed by pulseless VT, suspected venous gas embolism	Defibrillation and CPR in the prone position	ROSC, completion of surgery, extubation the following day, and discharge without consequence.
Reid and Appleton (1999) UK [26]	Case report “*Case of ventricular fibrillation in the prone position during back stabilisation surgery in a boy with Duchenne’s muscular dystrophy*”	Spinal surgery: Corrective surgery for progressive scoliosis	Boy 15 Y	VF	Left posterior thoracotomy and direct cardiac massage, defibrillation in prone position (2 discharges of 200 J)	ROSC after 40 min and admission to ICU intubated, discharged after 8 days from intensive care unit and continued admission to pediatrics. No neurological or organ deficits evident on admission to ICU.
Sutherland and Winter (1997) UK [32]	Case report “*Two cases of fatal air embolism in children undergoing scoliosis surgery*”	Spinal surgery: Case A: T1-sacral posterior spinal fusion with sublaminar wiring for progressive scoliosis; Case B: T2-sacral posterior spinal fusion with sublaminar wiring for progressive scoliosis	Case A: Girl 8 Y Case B: Girl 12 Y	Asystole, from massive venous embolism; 2 episodes of asystole, possible from venous embolism	For both cases, CPR was started in the prone position and then continued in the supine position	Intraoperative death of both patients. Pathology of case A:muscular dystrophy Pathology of case B:cerebral palsy.
Gueugniaud et al. (1995) France [27]	Case report “*Non-invasive contin uous haemodynamic and PETCO 2 monitoring during peroperative cardiac arrest*”	Spinal surgery: Correction of right dorsal scoliosis and left lumbar scoliosis	Boy 15 Y	PEA followed by asystole from probable myocardial ischemia	CPR in prone position and subsequent supination	ROSC, surgery postponed, discharge without neurological deficits.
Kelleher and Mackersie (1995) UK [28]	Case report “*Cardiac arrest and resuscitation of a 6-month old achondroplastic baby undergoing neurosurgery in the prone position*”	Cranial surgery: Decompression of the foramen magnum	Infant male 6 M	Multiple ventricular extrasystoles followed by bradycardia and Asystole (2 episodes) Suspected cause: venous gas embolism, estimated hemorrhage 1.1 L	CPR with two fingers of one hand in the prone position (for both episodes)	ROSC and admission to ICU, discharge at 7 days.
Tobias et al. (1994) USA [10]	Case report “*Intraoperative cardiopulmonary resuscitation in the prone position*”	Spinal surgery: Corrective surgery for progressive scoliosis	Boy 12 Y	Asystole, likely from major hemorrhage	CPR in the prone position	ROSC and ICU admission, uneventful postoperative course.
Loewenthal et al. (1993) France [29]	Case report “*Efficacité du massage cardiaque externe chez une patiente en décubitus ventral [Efficacy of external cardiac massage in a patient in the prone position]*”	Cranial surgery: Excision of posterior cranial fossa meningioma	Woman 53 Y	Asystole, likely from major hemorrhage	CPR in the prone position	ROSC, completion of surgery, admission to ICU.
Sun et al. (1992) Taiwan [30]	Case report “*Successful cardiopulmonary resuscitation of two patients in the prone position using reversed precordial compression*”	Cranial and spinal surgery: Case A: Emergency craniotomy for hematoma in posterior cerebral fossa following traffic accident; Case B: Emergency C3 decompressive laminectomy following a fall	Girl 14 Y Man 34 Y	PEA, from possible brainstem compression; PV, hypoxia from endotracheal tube obstruction	CPR in the prone position (for both cases), not performed defibrillation for the second case but cardiac massage for 6 min	ROSC of both cases, the former proceeded to surgery after a short ICU admission. In the second case, ROSC was obtained at 6 min of CPR and resolution of hypoxia, after which surgery was continued in the immediate, and then the patient was admitted to the ICU without deficit.
Kalenda and Greuter (1982) Holland [31]	Case report “*Sitting or prone? Another argument for the latter*”	Cranial surgery: Excision of an Ependymoma in the fourth ventricle of the brain	Infant male 1 Y	Cardiac arrest not specified	CPR in the prone position	ROSC, completion of surgery, admission to ICU.

## Supplementary Materials

The following supporting information can be downloaded at https://www.mdpi.com/article/10.3390/jcm14062044/s1: Supplementary file S1: Search strategy.
