# Supplementary material for: Prone Position and Cardiopulmonary Resuscitation in the Operating Room: A Scoping Review
Source: J Clin Med. 2025 Mar 17;14(6):2044. doi: 10.3390/jcm14062044 (PMC11942719; doi:10.3390/jcm14062044)
Supplement: Supplementary file 1 [file jcm-14-02044-s001.zip › jcm-3484808-supplementary.pdf]

Supplementary file 1: Search strategy

The search strategies for the chosen databases are as follows:

PubMed: "((cardiopulmonary Resuscitation OR resuscitation OR chest compression) AND (prone OR prone position OR prone positioning) AND (surgery OR operating room))"

ScienceDirect/Elsevier: "((prone) AND (cardiopulmonary resuscitation) AND (surgery))".

CINAHL: "((chest compressions) AND (prone position) AND (operating room))"

Cochrane: "((chest compressions) AND (prone position) AND (operating room))"

Scopus: (("cardiopulmonary resuscitation" OR resuscitation OR "chest compression") AND (prone OR "prone position" OR "prone positioning") AND (surgery OR "operating room"))

Web of Science: (("cardiopulmonary resuscitation" OR resuscitation OR "chest compression") AND (prone OR "prone position" OR "prone positioning") AND (surgery OR "operating room"))
